# ANFIS grid partition framework with difference between two sigmoidal membership functions structure for validation of nanofluid flow

**DOI:** 10.1038/s41598-020-72182-5

**Published:** 2020-09-21

**Authors:** Mahboubeh Pishnamazi, Meisam Babanezhad, Ali Taghvaie Nakhjiri, Mashallah Rezakazemi, Azam Marjani, Saeed Shirazian

**Affiliations:** 1 Institute of Research and Development, Duy Tan University, Da Nang, 550000 Vietnam; 2 The Faculty of Pharmacy, Duy Tan University, Da Nang, 550000 Vietnam; 3 Department of Chemical Sciences, Bernal Institute, University of Limerick, Limerick, Ireland; 4 Department of Energy, Faculty of Mechanical Engineering, South Tehran Branch, Islamic Azad University, Tehran, Iran; 5 Department of Petroleum and Chemical Engineering, Science and Research Branch, Islamic Azad University, Tehran, Iran; 6 Faculty of Chemical and Materials Engineering, Shahrood University of Technology, Shahrood, Iran; 7 Department for Management of Science and Technology Development, Ton Duc Thang University, Ho Chi Minh City, Vietnam; 8 Faculty of Applied Sciences, Ton Duc Thang University, Ho Chi Minh City, Vietnam; 9 Laboratory of Computational Modeling of Drugs, South Ural State University, 76 Lenin prospekt, 454080 Chelyabinsk, Russia

**Keywords:** Software, Computational science, Structure prediction, Computational chemistry

## Abstract

In this study, a square cavity is modeled using Computational Fluid Dynamics (CFD) as well as artificial intelligence (AI) approach. In the square cavity, copper (Cu) nanoparticle is the nanofluid and the flow velocity characteristics in the x-direction and y-direction, and the fluid temperature inside the cavity at different times are considered as CFD outputs. CFD outputs have been assessed using one of the artificial intelligence algorithms, such as a combination of neural network and fuzzy logic (ANFIS). As in the ANFIS method, we have a non-dimension procedure in the learning step, and there is no issue in combining other characteristics of the flow and thermal distribution beside the x and y coordinates, we combine two coordinate parameters and one flow parameter. This ability of method can be considered as a meshless learning step that there is no instability of the numerical method or limitation of boundary conditions. The data were classified using the grid partition method and the MF (membership function) type was *dsigmf* (difference between two sigmoidal membership functions). By achieving the appropriate intelligence in the ANFIS method, output prediction was performed at the points of cavity which were not included in the learning process and were compared to the existing data (the results of the CFD method) and were validated by them. This new combination of CFD and the ANFIS method enables us to learn flow and temperature distribution throughout the domain thoroughly, and eventually predict the flow characteristics in short computational time. The results from AI in the ANFIS method were compared to the ant colony and fuzzy logic methods. The data from CFD results were inserted into the ant colony system for the training process, and we predicted the data in the fuzzy logic system. Then, we compare the data with the ANFIS method. The results indicate that the ANFIS method has a high potentiality compared to the ant colony method because the amount of R in the ANIFS system is higher than R in the ant colony method. In the ANFIS method, R is equal to 0.99, and in the ant colony method, R is equal to 0.91. This shows that the ant colony needs more time for both the prediction and training of the system. Also, comparing the pattern recognition in the two systems, we can obviously see that by using the ANFIS method, the predictions completely match the target points. But the other method cannot match the flow pattern and velocity distribution with the CFD method.

## Introduction

Natural convection heat transfer of a heated enclosure has widespread application in industries dealing with fluids. Its application includes solar collectors, thermal storage systems, building thermal design, aviation thermal management, heat dissipation from electronic devices, air conditioning, heaters, chemical processing tools, lubrication, and drying technologies^1–3^. To improve the heat transmission features inactively, utilizing fluid with high thermal conductivity is usual. Furthermore, rich heat dissipation properties can be achieved by using material that has a higher surface area^4–6^. There are also several research studies focusing on the effect of physical parameters on the nanofluid system and the thermal distribution of nanofluid in physical problems^7–10^.

In thermal engineering uses, the nano-sized additives are utilized in the base fluid with a lower thermal conductivity^11^. Better thermal conductivity characteristics of base fluid are obtained through the higher thermal conductivity of nanoparticles, even for the small quantity of metallic or nonmetallic additive particles, including Al_2_O_3_, Cu, Ag, Au, CuO, SiO_2_, TiO_2_ with an average particle size less than 100 nm^12–16^.

Investigating thermal structures employing experimental physics involves some drawbacks as well as limitations, although it is constantly a useful and important trend^17,18^. The challenging determination of the role of different physical phenomena to the system effectiveness is one of the disadvantages. Furthermore, high-cost tests regularly make an obstruction for performing comprehensive experimental investigations^19–22^. Simulation and modeling the optimal system progressively provide an appropriate substitute for the tests. Cubic Interpolation Particle (CIP) among all numerical methods has been currently developed for modeling the nanoparticles. In addition to this numerical model, machine learning techniques have been mixed with numerical or experimental data for predicting the multiphase flow. In about one decade, some researchers^23,24^ have combined the CFD method with machine learning algorithms to predict the fluid flow, particularly multiphase flow and heat transfer problems^25,26^. They examined different tuning parameters to achieve the best accuracy of the model in predicting the flow^27–29^.

For predicting the process of engineering, numerous machine learning techniques exist^30–33^. Intelligent methods and soft numerical algorithms such as neural networks^34–37^ and support vector machines^35–37^ are frequently used in the prediction of process engineering in industries or physics in real-life applications. The combination of neural cells and fuzzy structure called ANFIS is usually recommended for the prediction process^38^, and it is capable of directing complex associations. A smart approach is provided by this model to estimate the complex mechanisms in engineering^39^. Regulating the robotic movements in hazardous circumstances is an appropriate instance that can be mentioned here. Consequently, such techniques are appropriate to control the robots ant time that hazardous reactions can harm people^40^. Combination of machine learning and experimental or numerical outputs enables us to accelerate the optimization of process engineering, particularly when we require to run many CFD simulations to optimize the process^41–43^.

As far as the numerical method for solving of the CFD could take much time from researchers and engineers, and understanding of the big datasets is difficult, the machine learning method can be combined with CFD. Therefore, the results from CFD data could be used for the prediction of the process in CFD form. The results also could use the CFD datasets for a better understanding of the engineers. On the other hand, the conventional methods are weak for learning and understanding of the CFD data. Furthermore, they do not have the ability to train the matrices with different dimensions in order to provide a good understanding of the dataset for the engineers. AI has the ability to create mathematical correlations between the inputs and outputs, which could be beneficial in the training of a process. In previous studies, different processes have been studied in AI and CFD methods. The combination of the mentioned methods was used in different processes, including bubbly flow in the bubble column reactors, and nanofluid characteristics. In the current study, we used the ANFIS method and different membership functions to have the accuracy of the ANFIS method for creating the best prediction in our system. After the prediction in the ANFIS method, we also used the ACO method to complete the training process with this method, and the prediction completed by using the fuzzy logic system. After the prediction, ANFIS and ACO methods were compared with each other to study the accuracy of the ANFIS with other machine learning methods.

In this study, the liquid flow pattern and temperature distribution in a square cavity are calculated based on the CFD method. Different fluid parameters are obtained as the output of the CFD method. The flow characteristics include the flow pattern in different spaces, and the fluid temperature inside the cavity. In particular, grid partition with *dsigmf* as the membership function type was used for data classification, and by altering the structure of membership for neural cells, the influence of this parameter on the ANFIS intelligence was evaluated.

## CFD method

**Figure 1:**
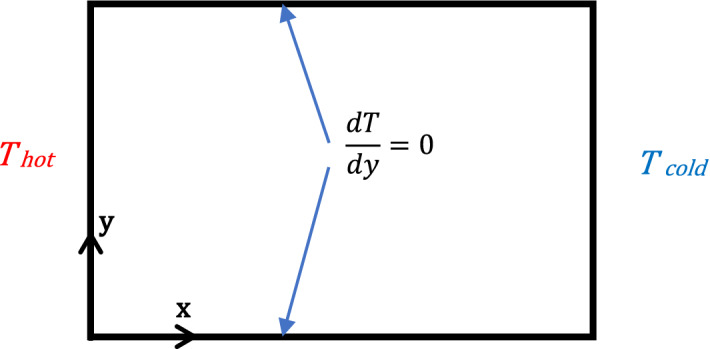
Representation of the square space and its boundary conditions^17^.

A square surrounded area along with its physical specifications is shown in Fig. 1. The temperatures in upright left and right solid sections of the empty space are maintained constant (see Fig. 1), and their particular dimensionless values are equivalent to one. The right-hand side wall is colder than the left. The adiabatic assumption (no heat transfer) is implemented to the bottom and top walls of the simulation geometry. The nanofluid comprises of Cu particles suspended in water. Cubic-Interpolated Pseudo-particle (CIP)^17^ has been used to simulate the flow pattern and temperature distribution in the square shape cavity.

To start the model development, vorticity equation may be expressed as^17^:

1 $$ \frac{\partial \omega }{{\partial t}} + \frac{\partial }{\partial x}\left( {\omega \frac{\partial \psi }{{\partial y}}} \right) - \frac{\partial }{\partial y}\left( {\omega \frac{\partial \psi }{{\partial x}}} \right) = \frac{{\mu_{nf} }}{{\rho_{nf} }}\left( {\frac{\partial \omega }{{\partial x^{2} }} + \frac{\partial \omega }{{\partial y^{2} }}} \right) + \frac{{\left( {\phi \rho_{s} \beta_{s} + \left( {1 - \phi } \right)\rho_{f} \beta_{f} } \right)}}{{\rho_{nf} }}g\left( {\frac{\partial T}{{\partial x}}} \right) $$

Also, the energy equation^17^:

2 $$ \frac{\partial T}{{\partial t}} + \frac{\partial }{\partial x}\left( {T\frac{\partial \psi }{{\partial y}}} \right) - \frac{\partial }{\partial y}\left( {T\frac{\partial \psi }{{\partial x}}} \right) = \frac{\partial }{\partial x}\left( {\alpha_{nf} \frac{\partial T}{{\partial x}}} \right) + \frac{\partial }{\partial y}\left( {\alpha_{nf} \frac{\partial T}{{\partial y}}} \right) $$

The thermal diffusivity is calculated as^17^:

3 $$ \alpha_{nf} = \frac{{k_{nf} }}{{(\rho c_{p} )nf}} $$

The density of a fluid in the square shape domain that has floating particles is calculated using^17^:

4 $$ \rho_{nf} = \left( {1 - \phi } \right)\rho_{f} + \phi \rho_{s} $$

In which, the parameters $\phi$, $\rho_{f}$, and $\rho_{s}$ are the volumetric fraction of the floating particles, density of the particles, and the density of the pure fluid, successively^17^.

5 $$ \mu_{nf} = \frac{{\mu_{f} }}{{\left( {1 - \phi } \right)^{2.5} }} $$

The formulation for effective stagnant thermal conductivity of fluid mixture was primarily suggested by Wasp^44^ and also used in^17^:

6 $$ \frac{{k_{nf} }}{{k_{f} }} = \frac{{k_{s} + 2k_{f} - 2\phi (k_{f} - k_{s} )}}{{k_{s} + 2k_{f} + \phi (k_{f} - k_{s} )}} $$

The CIP model is implemented to derive the advection term in order to calculate vorticity. Additional details concerning the numerical calculations are given in^17^.

The Nusselt number is calculated for a mixture of Nanoparticles in the fluid, and it is controlled by some thermal parameters. The local Nusselt number for the nanofluid is given by^17^:

7 $$ Nu_{y} = - \left( {\frac{{K_{nf} }}{{K_{f} }}} \right)\frac{\partial \theta }{{\partial X}}\;{\text{and}}\;Nu_{x} = - \left( {\frac{{K_{nf} }}{{K_{f} }}} \right)\frac{\partial \theta }{{\partial Y}} $$

The Nusselt number of the nanofluids is a function of thermal characteristics, such as thermal conductivity and heat capacitance of both liquid and particles (nanomaterials). We have measured the Nusselt number numerically through the cavity domain. More specifically, the average Nusselt is calculated based on the integral over all computing nodes in x-direction and y-direction, and the average Nusselt number is derived from^23^:

8 $$ Nu_{avg} = \int_{0}^{{l_{x} }} {Nu_{y} dy} + \int_{0}^{{l_{y} }} {Nu_{x} dx} $$

The nanofluid is created by using water, and the water includes Cu with uniform volume fraction, and we use a square shape domain to simplify the solving. The fluid in the cavity is incompressible and Newtonian. Moreover, there is no viscosity dissipation, and the flow regime is laminar. The current study is two-dimensional, and the CIP method is used for minimizing the numerical diffusion for high order Naiver Stokes equation. The characteristics of the nanofluids used in the fluid is sphere shape particles of Cu in the water.

The characteristics of the nanoparticles are according to the following features:

The density of Cu is 8,933 kg/m^3^, Specific heat at constant pressure (C_P_) for Cu is 385 (j/kg K), thermal conductivity is 401 (w/m k) and Electrical conductivity is 5.96 × 10^7^.

## ANFIS method

**Figure 2:**
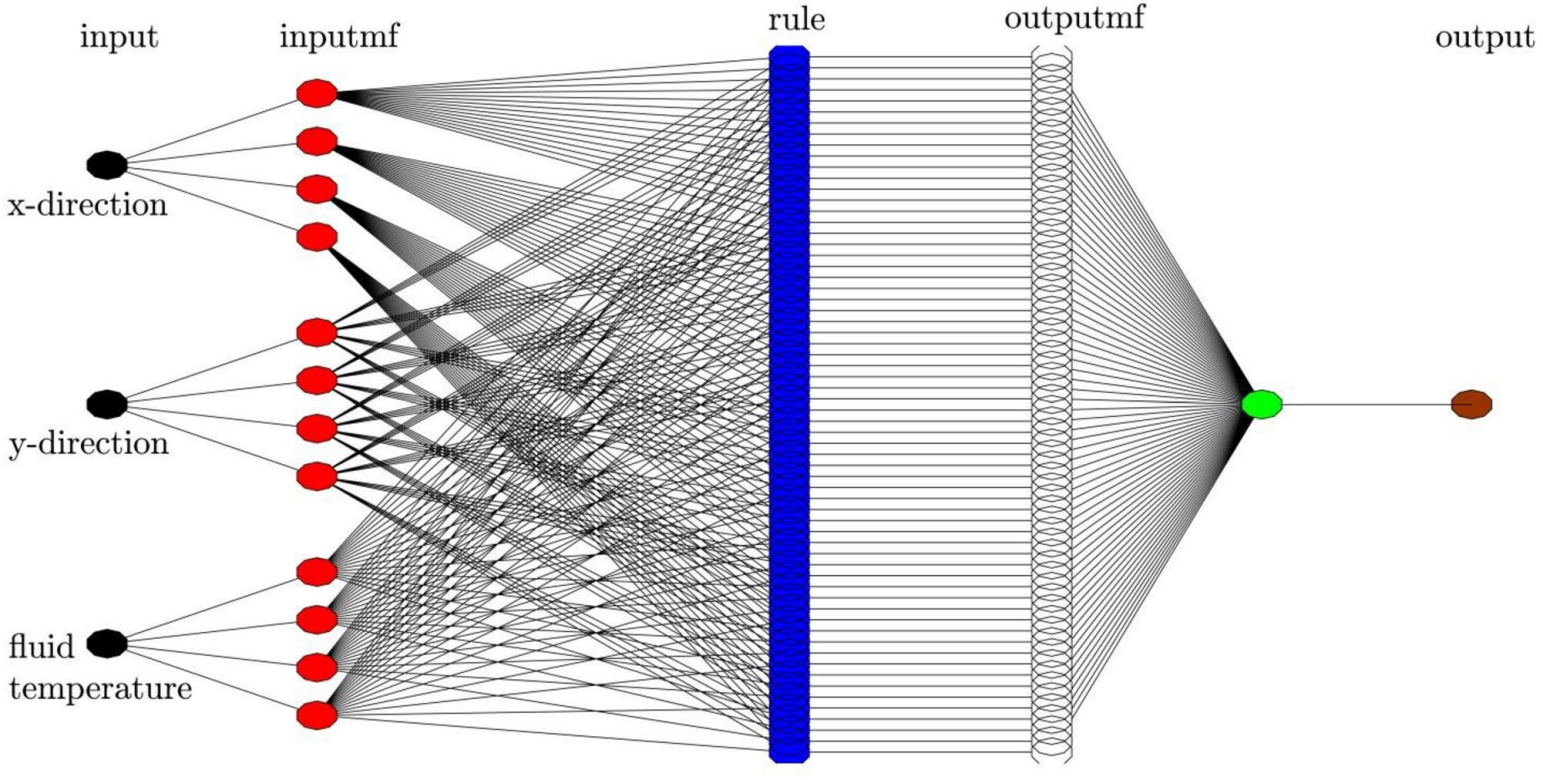
ANFIS structure with three inputs, number of inputs MFs = 4, type of MFs = *dsigmf*.

ANFIS is considered as a fuzzy extrapolation system to forecast the behavior of the nonlinear and complex system precisely^45,46^. Various kinds of fuzzy reasoning exist implementing the If–Then rules suggested by Takagi and Sugeno in ANFIS systems^47^. The scheme of the utilized ANFIS technique to predict the hydrodynamic properties is provided in Fig. 2 in the cavity. In this work, the location of CFD elements (x and y coordinate), as well as temperature distribution in the cavity, are considered as input parameters of the learning method. However, for the output parameter, the flow characteristics such as velocity distribution is used in the training method. Detailed descriotion of ANFIS method can be found in our previous publications^23,24,27–29,46,48–50^.

## Results and discussion

It has been recognized that addition of nanoparticles inside the liquid leads to the increase of heat transfer and improvement of the efficiency of a system. As previous studies have shown, increasing the actions of the nanoparticles in a heating system could change the heating potentiality and increase the heat transfer of the system. In this research, we study that AI has the potentiality to predict the data relating to nanofluid. To do so, we added the nanofluid in the CFD, and after the training of the CFD dataset, we introduce the data set in the intelligence algorithm to see whether AI could predict CFD data when systems included nanoparticles.

In this study, the temperature distribution, as well as flow field in a square shape cavity, are calculated using the CFD method. Different fluid characteristics are obtained as the output of the CFD method, such as flow pattern in the x-direction and y-direction, and the thermal fluid distribution inside the cavity. In the present study, three inputs were used in the learning process of the ANFIS method, including the coordinates in the x-direction as the first input and coordinates in the y-direction as the second input and the temperature of the fluid inside the cavity as the third input. Also, the fluid velocity in the x-direction (u) was considered as the output. In the learning stage, the maximum iteration was considered to be 800, P = 65% (P represents the percentage of the total data which has been included in the training process). In the training process, 65% of the data was involved in the testing stage, and 100% of the data were considered for validation of the training stage. The Grashof number (Gr), which is a dimensionless number in fluid dynamics and heat transfer, was considered to be 11,000, and *φ* = 5% (*φ* represents the percentage of nanoparticles in the fluid showing the volume of nanoparticles in the liquid). Also, the Grid partition method was used in this study to classify the data and *dsigmf* (Difference between two sigmoidal membership functions) was considered as the type of membership function (MF).

**Figure 3:**
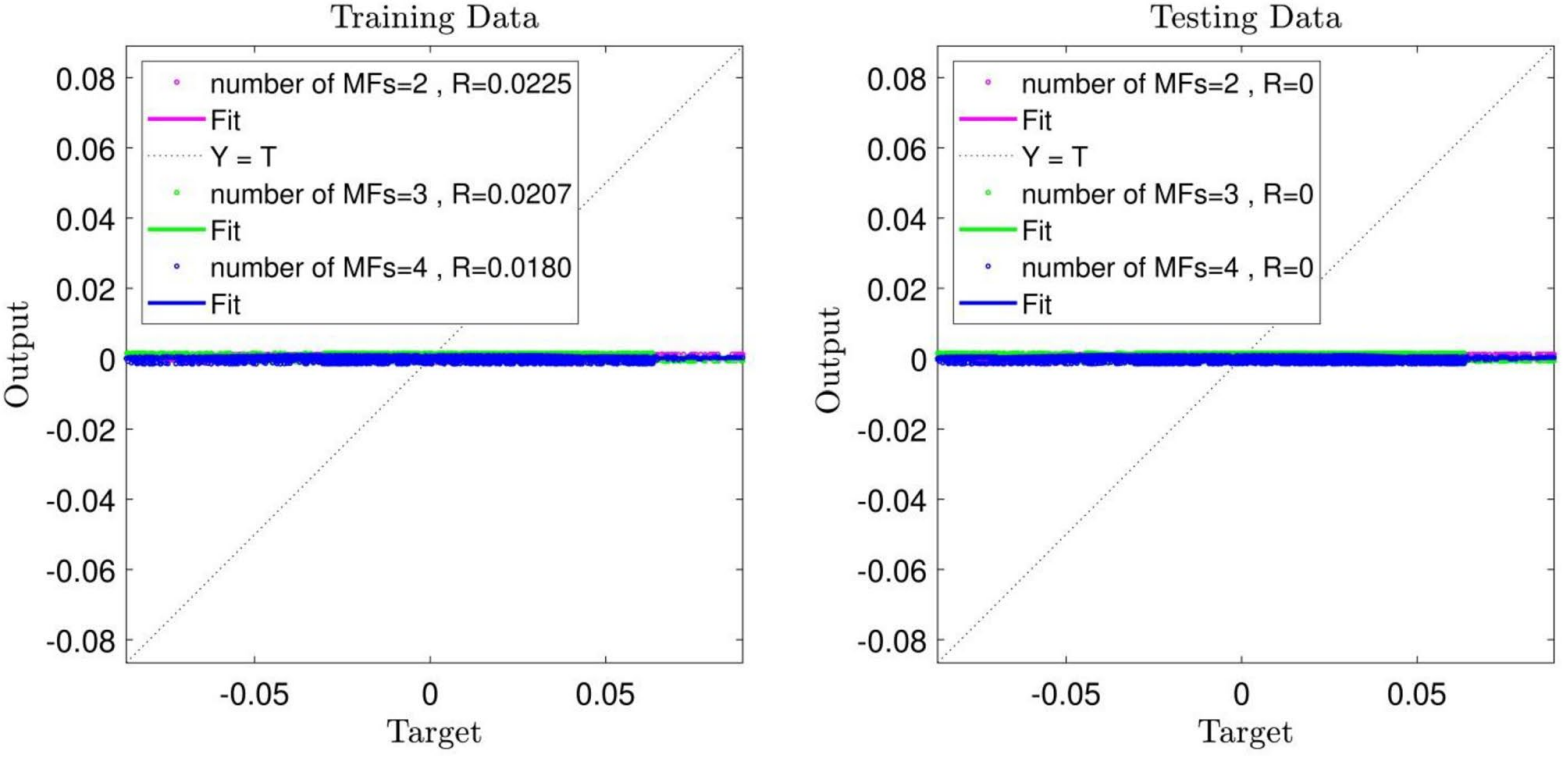
ANFIS training and testing process: 1 input, Max Iteration = 800, type of MFs = *dsigmf*, number of inputs MFs = (2, 3, 4).

With the aforementioned assumptions and by considering an input in the ANFIS method learning process (x-direction coordinates) and an output (fluid velocity in the x-direction (u)), the learning process, which includes the training and testing stages, was separately performed for a number of MFs (MF = 2, 3, 4). As shown in Fig. 3, the value of Regression (R) in all of the learning processes equals zero. Changing the number of MFs had no impact on the increase of ANFIS intelligence.

**Figure 4:**
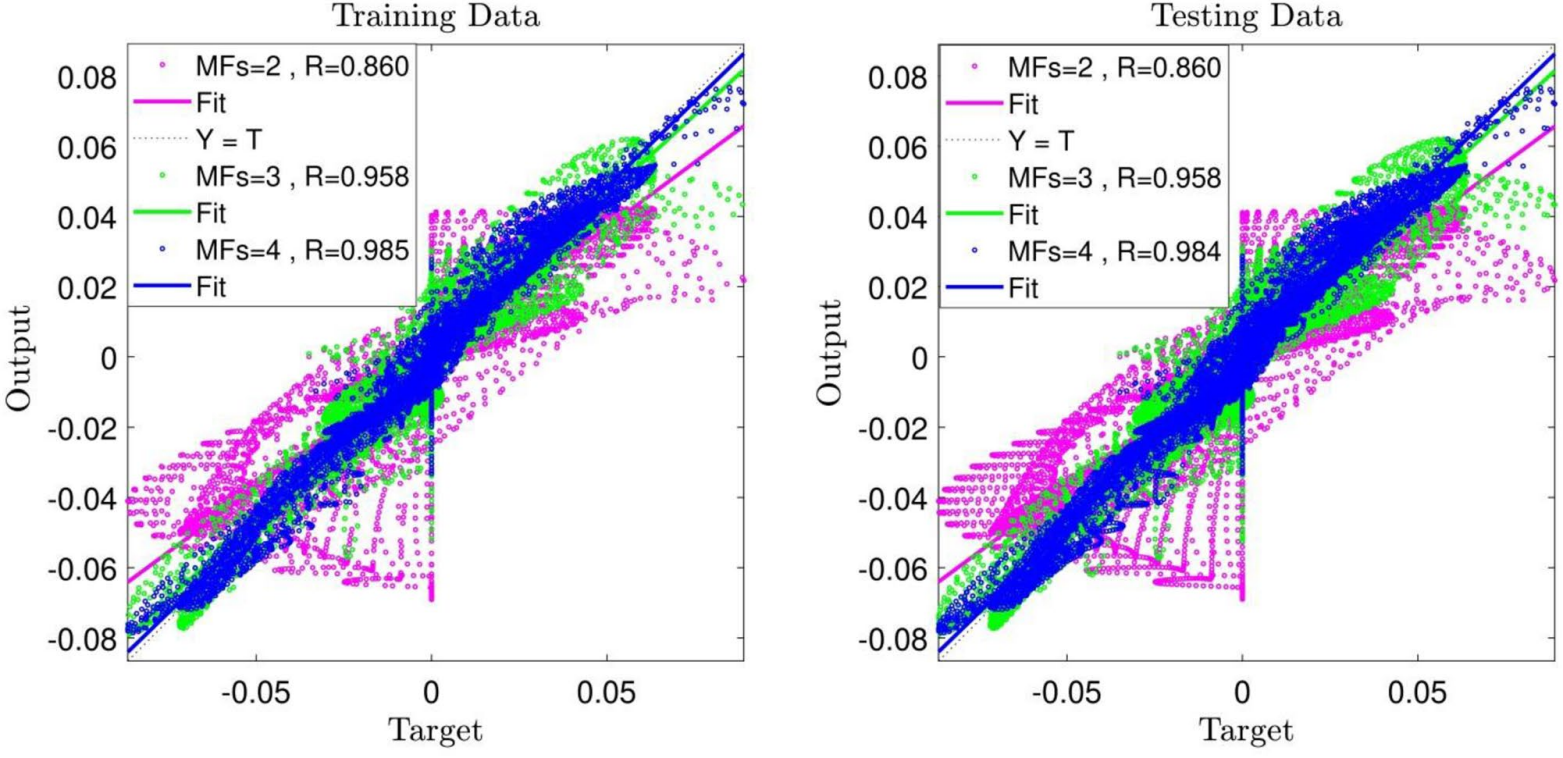
ANFIS training and testing process: 2 inputs, Max Iteration = 800, type of MFs = *dsigmf*, number of inputs MFs = (2, 3, 4).

Afterward, to promote the model, the No. of inputs was increased, and the coordinates in x-direction and y-direction were considered as the inputs of ANFIS, while the fluid velocity in the x-direction (u) was considered as output. Training/testing procedures were performed for two MFs. As shown in Fig. 4, a good improvement in the intelligence of the system was achieved and the value of R increased to 0.86. The number of MFs was changed to evaluate the increase in the system intelligence; the No. was increased to 3, and the learning process was carried out. The value of R was increased to 0.95, which indicates the positive effect of this change; hence the number of MFs was increased to 4, resulting in an R-value of 0.98.

**Figure 5:**
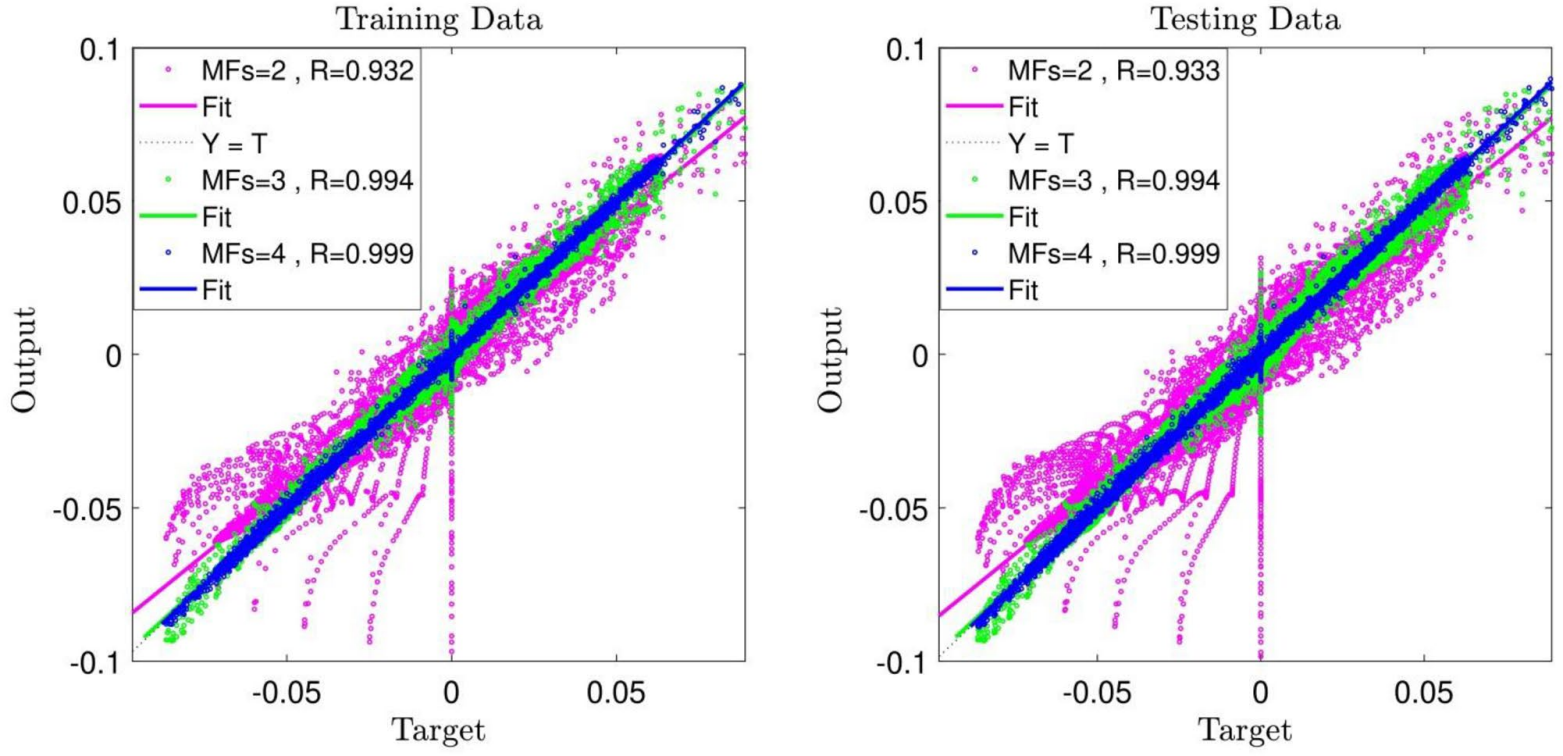
ANFIS training and testing process: 3 inputs, Max Iteration = 800, type of MFs = *dsigmf*, number of inputs MFs = (2, 3, 4).

Subsequently, the number of inputs was increased from 2 to 3, the x-direction and y-direction coordinates and the fluid temperature were considered as the inputs of the learning process and the fluid velocity in the x-direction (u) was considered as output. The training/testing stages were carried out for a number of MFs; the obtained value of 0.93 for R is indicative of a 7% increase in the system intelligence compared to the similar conditions in which two inputs participated in the learning stage. After that, the number of MFs was increased to 3 and the learning process was performed once more. According to Fig. 5, the increase of the R value to 0.99 indicates achieving high intelligence for the ANFIS method.

**Figure 6:**
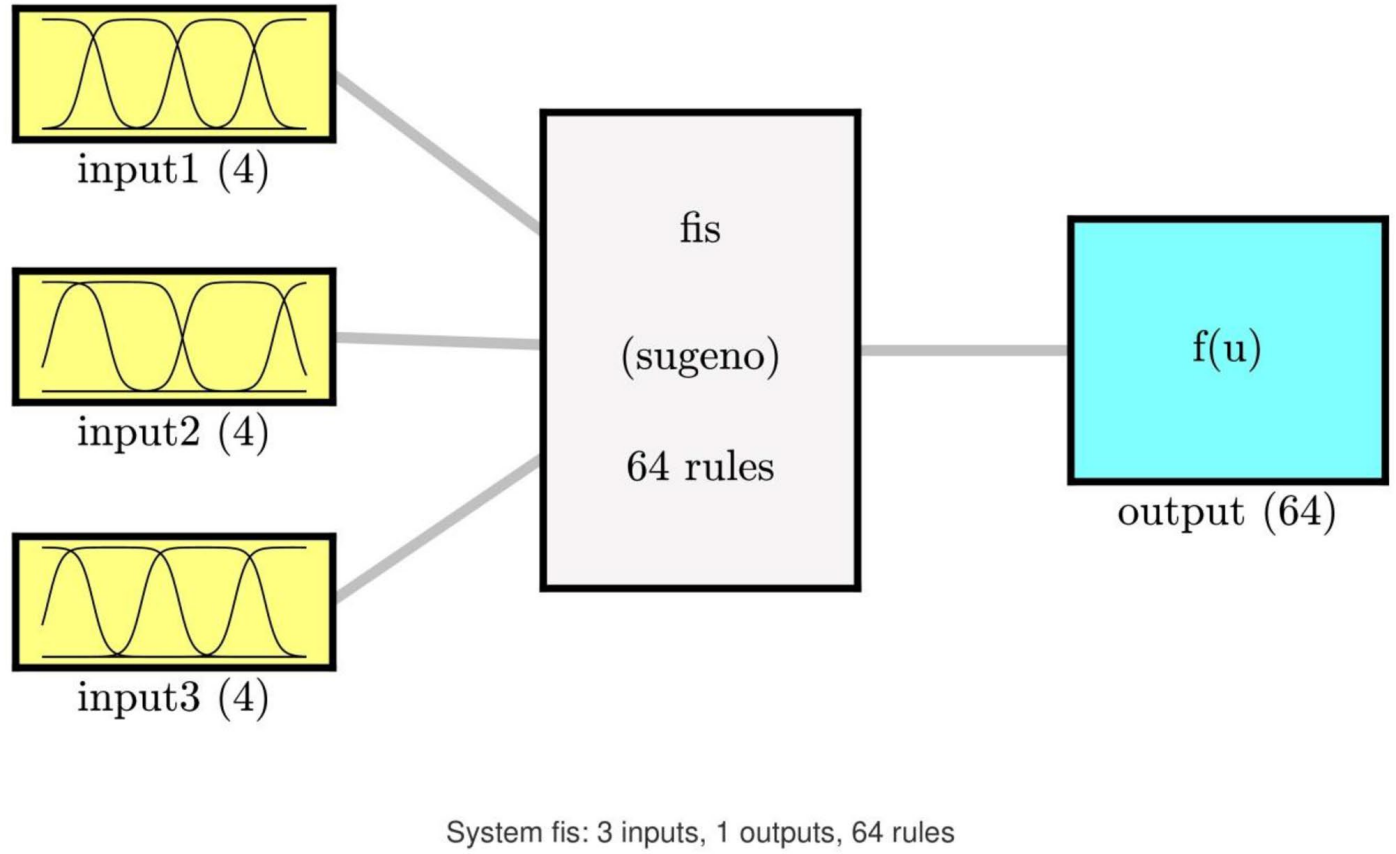
System FIS: 3 inputs, Max Iteration = 800, type of MFs = *dsigmf*, number of inputs MFs = (4), number of rules = 64.

By increasing the number of MFs to 4 and performing the learning process, we observe a value of 0.999 for R, showing that the system reached complete intelligence. Using this suitable intelligence, we can predict nodes that are not present in the training stage, which shows the functionality and practicality of using the ANFIS method^23^. In Fig. 6, the schematic view of the ANFIS system is observed, which illustrates the creation of 4 rules for each individual input and 64 rules for output.

**Figure 7:**
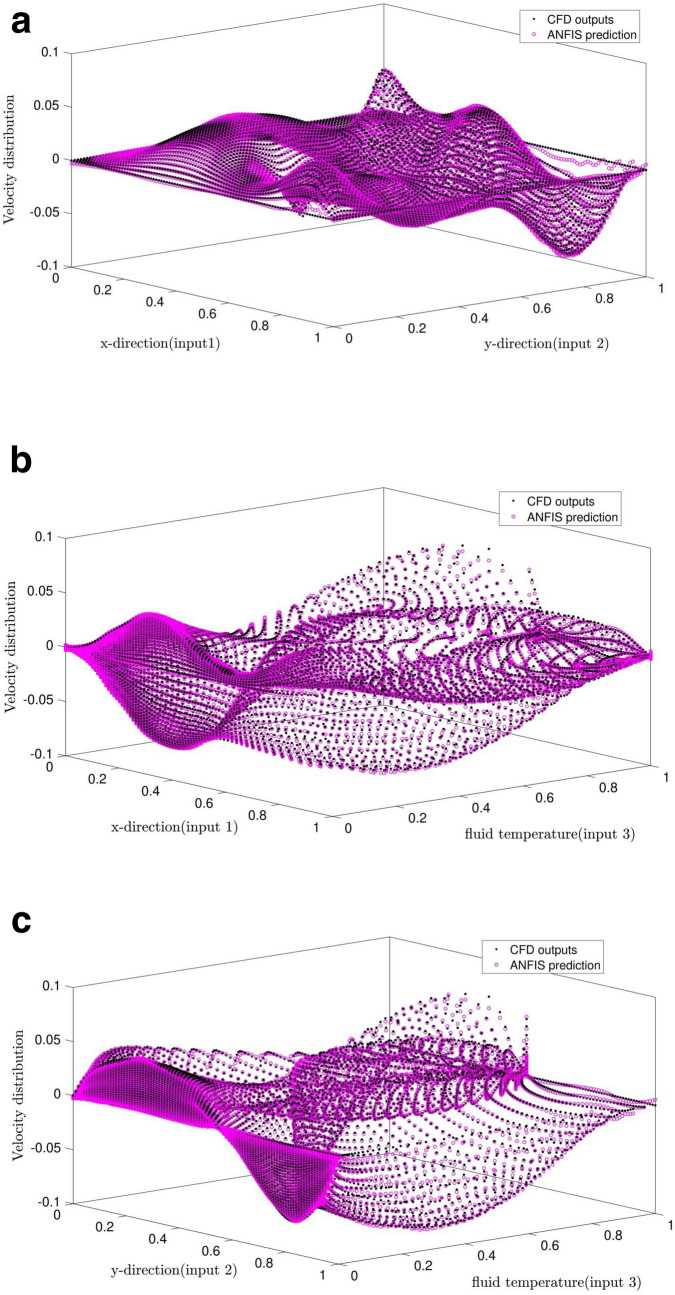
(**a**) Validation of ANFIS (inputs 1 and 2), Max Iteration = 800, type of MFs = *dsigmf*, number of inputs MFs = (4). (**b**) Validation of ANFIS (inputs 1 and 3), Max Iteration = 800, type of MFs = *dsigmf*, number of inputs MFs = (4). (**c**) Validation of ANFIS (inputs 2 and 3), Max Iteration = 800, type of MFs = *dsigmf*, number of inputs MFs = (4).

By using ANFIS intelligence, we can obtain more information. According to Fig. 7, the comparison of the CFD and ANFIS results validates the ANFIS predictions and CFD data. The combination of ANFIS and CFD creates the ability to predict points that are not present in the learning process, as well as predictable points that are not created by CFD, which is a very useful feature for combining the ANFIS and CFD methods. The results indicate a positive effect of using *dsigmf* as a membership function and selecting the grid partition as a clustering type to achieve the full ANFIS intelligence.

In the current research paper, we study the comparison of results between the ANFIS and ant colony methods. In our study, the ant colony method was used for the training of the system, and the fuzzy structure system was used for the prediction of our dataset.

**Figure 8:**
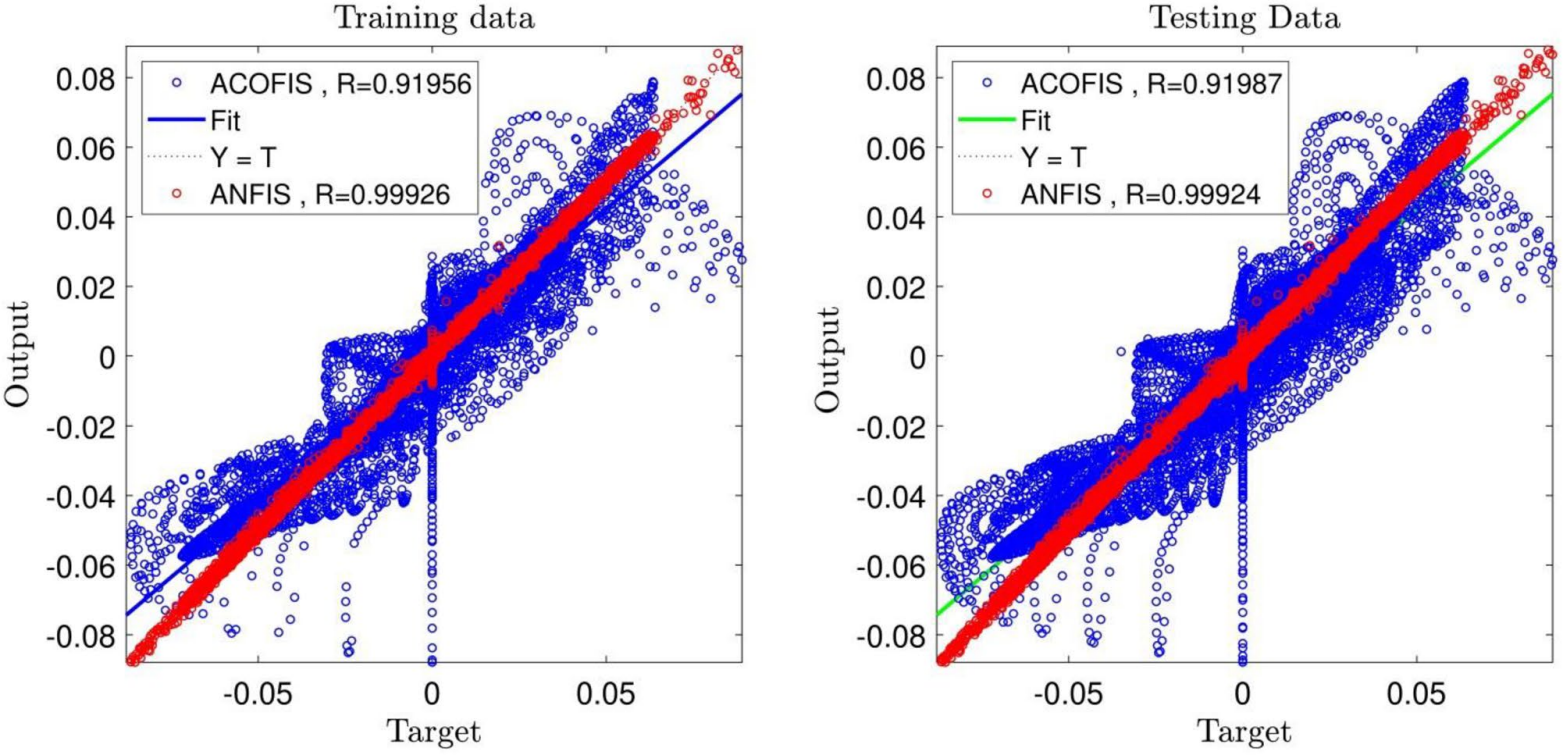
Comparison of correlation coefficient(R) between the best result of ANFIS method and Ant Colony Optimization based Fuzzy Inference System (ACOFIS).

As shown in Fig. 8, the ANFIS method has high potentiality for the flow prediction in the square shape domain. The R criterion for this method is equal to 0.99, and the criteria for the ant colony is equal to 0.92. This shows that ANFIS has high accuracy in prediction, meaning that neural networks have high potentiality for the training of the data compared to the ACO method. For a better comparison of predictability of the methods, we study the methods in pattern recognition comparison. We compare the CFD and velocity distribution patterns for ANFIS and ACO methods, and the two methods were compared to CFD data to see which method can predict the patterns in the domain.

**Figure 9:**
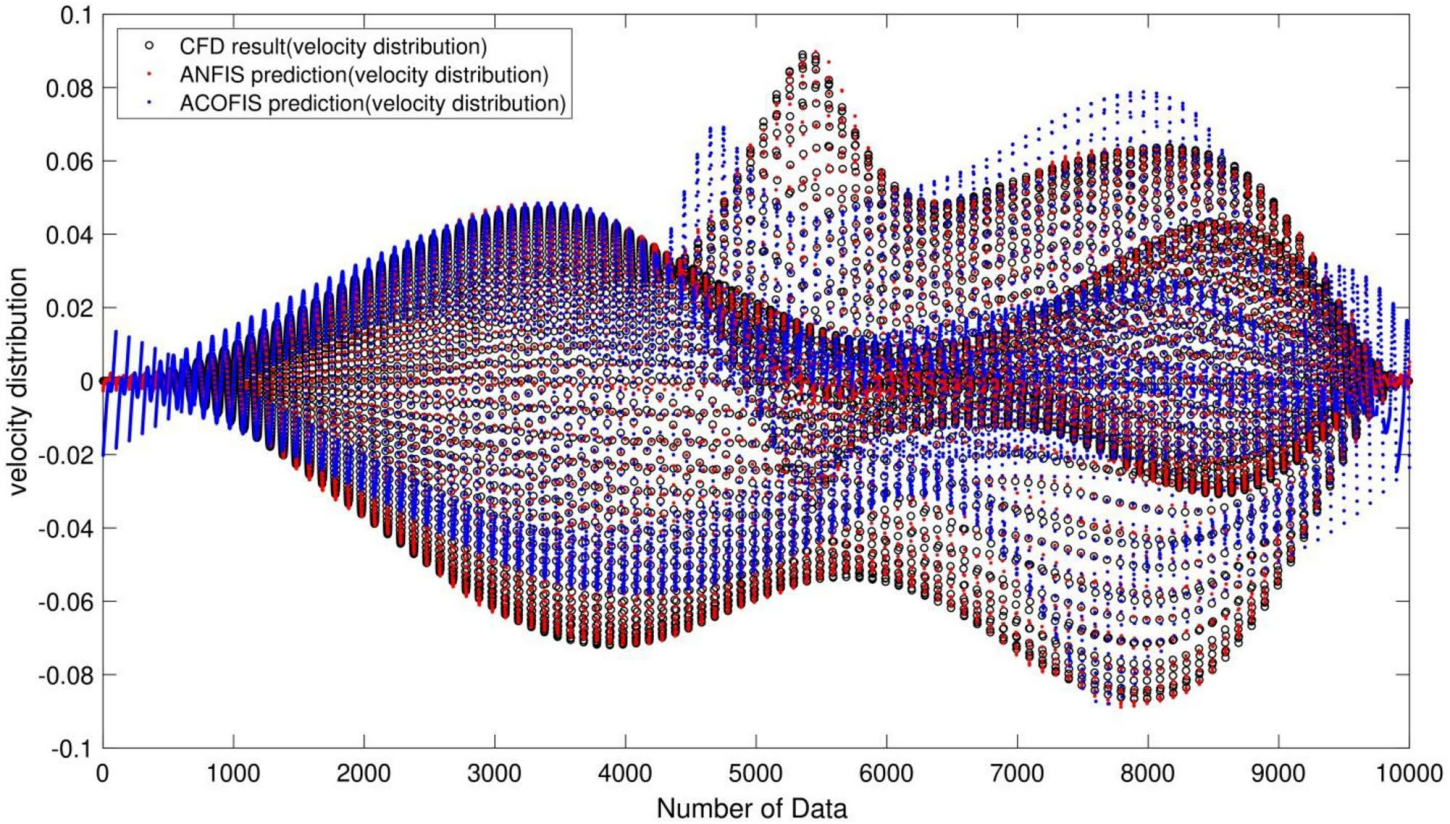
Comparison of velocity distribution between the best result of ANFIS and ACOFIS methods.

As shown in Fig. 9, ANFIS has high potentiality for predicting the flow pattern. However, ACO cannot predict the pattern well, and the predicted data does not match the CFD data meaning the data differs significantly with the CFD method. Due to the comparison of the methods by using R criteria, the ANFIS method has a lower error. The two methods can show us the flow inside the cavity. They also show the flow streamline in the middle of the domain, indicating zero velocity distribution in the middle of the square shape, and by moving further from the middle of the domain, the fluid velocity could increase.

## Conclusions

In this study, nanofluid was simulated in a cavity by using CFD, and the fluid characteristics were studied by combining one of the methods of artificial intelligence (ANFIS) with CFD. In this study, one of the data clustering methods called Grid partition was used, and the number of MFs was considered as one of the grid partition variables. The changes in the number of MFs and the number of inputs were considered as the most important parameters influencing the ANFIS intelligence enhancement, which had an appropriate impact on the enhancement of ANFIS intelligence. The use of *dsigmf* as one of the varieties of MFs in this study has been effective in producing high-level intelligence that results in high accuracy of prediction.

One of the huge applications of the current research study is that repetitive solving of the CFD method, we could delete them and use AI instead. The AI method is a substitution for CFD to optimize the process, and it can avoid repetitive solving in the optimization process. On the other hand, one of the best applications that this method could create with itself is that the AI method has the ability to create mathematical correlations that can examine the physical phenomena partially. Mathematical correlations could run as solvers in the methods and software packages to decrease the role of CFD in solving the mechanical and chemical processes. When AI methods complete the training in different dimensions of the data, the methods could create a good understanding of the data. They can also create good relationships between the inputs and output, which are very difficult to be understood by conventional methods. If we want to understand only the CFD data, it will be very time-consuming. But the AI methods help us to study the effect of each input and output parameters on each other. So we can have a better understanding of the process. The current study shows us that AI could have the ability of prediction for thermal and flow characteristics in a square shape domain. When the nanofluid is used in the domain, we used AI for two different methods, which are ANFIS and ACO methods plus the fuzzy logic system.

Comparing two methods for the prediction and flow characteristics, the ANFIS method has a high ability in the prediction of the data, and the flow characteristics in the domain. Nevertheless, the other method, could not predict some local points in the domain, while it can complete the prediction to some extent. So the prediction ability of ACO is lower than the ANFIS method. The two methods can show us the flow in the domain. Also, they can show the flow streamline in the domain, and in the middle of the domain, a zero velocity distribution is seen. By moving further from the middle of the domain, the velocity of points in the domain increases. For future studies for the purpose of prediction of nanofluid in a domain, we can use a variety of methods to compare their abilities with each other. For instance, we can use deep learning to see how the prediction happens via this method. We can also consider different operation conditions to increase the ability of training in machine learning, and therefore, the system can consider different conditions for the prediction.

## Abbreviations

*c*_*p*_: Specific heat capacity of the system
*g*: Gravity
*H*: Height of domain
*K*: Thermal conductivity in the cavity
*Nu*: Nondimensional Nusselt number
*t*: Time
*T*: Temperature in the domain
*u, v*: Velocity in different directions
*U,V*: Dimensionless velocity
*x, y*: Coordinates
*X, Y*: Dimensionless coordinates

## Greek symbols

*α*: Thermal diffusivity in the domain
*ϕ*: Solid volume fraction
*ν*: Kinematic viscosity
*ω*: Vorticity of the fluid flow
*ρ*: Density of fluid
*μ*: Dynamic viscosity

## Subscripts

*f*: Fluid in the cavity
*nf*: Nanofluid
